# 3D‐Printed Ultra‐Thin Non‐Prep Lithium Disilicate Veneers: A Proof‐of‐Concept Clinical Case

**DOI:** 10.1111/jerd.13427

**Published:** 2025-01-29

**Authors:** Alexey Unkovskiy, Florian Beuer, Jeremias Hey, Daniel Bomze, Franziska Schmidt

**Affiliations:** ^1^ Department of Prosthodontics, Geriatric Dentistry and Craniomandibular Disorders Charité–Universitätsmedizin Berlin Berlin Germany; ^2^ Department of Dental Surgery Sechenov First Moscow State Medical University Moscow Russia; ^3^ Department of Prosthodontics University of Halle Halle an der Saale Germany; ^4^ Lithoz GmbH Vienna Austria

**Keywords:** 3D‐printing, additive manufacturing, CAD/CAM, laminate veneers, LCM technology, lithium disilicate ceramics

## Abstract

**Objective:**

To test the clinical application of LCM‐printed lithium disilicate veneers.

**Clinical Considerations:**

A female patient with tooth wear was scanned with intraoral scanner (Prime Scan, Dentsply Sirona). The six non‐prep veneers were designed (DentalCAD, Exocad) and printed from lithium disilicate with an LCM‐printer with the thickness of 0.1–0.2 mm (CeraFab System S65 Medical, Lithoz GmbH). The green parts after printing were cleaned of the excess slurry and debindered in a furnace (Nabertherm, Lilienthal) until 430°C with a dwell time of 5 h to remove all polymeric binder. The resulting parts were sintered in an oven (Programat P510, Ivoclar Vivadent AG) at a temperature of 900°C. The supports were removed with a dental technician handpiece (Perfecta 900, W & H). The final restorations were stained (e‐max Ceram, Ivoclar Vivadent AG) by a master dental technician.

**Conclusions:**

The six LCM‐printed ultra‐thin non‐prep lithium disilicate veneers were tried‐in on the patient using a try‐in glycerin gel (Ivoclar Vivadent, Schaan, Lichtenstein) and demonstrated excellent fit and esthetics. LCM technology enabled the production of ultra‐thin non‐prep lithium disilicate veneers with layer thicknesses of down to 0.1 mm.

**Clinical Significance:**

The 3D‐printing of ultra‐thin non‐prep veneers is technically feasible and provides an adequate clinical outcome. Additive manufacturing of ultra‐thin non‐prep lithium disilicate veneers may pose a valid alternative to conventional and subtractive manufacturing.

## 1 Introduction

Dentistry is evolving toward the reduction of invasiveness [1]. In terms of prosthodontics this means the preservation of as much tooth structure as possible [2]. One of the potential materials for thin and minimally‐ or non‐invasive restorations is ceramics [3]. Traditionally, such ceramic restorations have been predominantly created through the heat pressing method [4]. The introduction of computer‐aided design and manufacturing (CAD/CAM) in dentistry enabled the production of ceramic restoration by milling technologies [5, 6, 7]. However, subtractive manufacturing does not offer complete freedom of design, as certain areas may be inaccessible to a milling cutter and milling of fragile ultra‐thin restorations may be hardly feasible [8, 9]. In this terms, additive manufacturing, with its layer‐by‐layer approach, outperforms subtractive methods [6, 10]. In the last years, additive manufacturing of zirconia and lithium disilicate using lithography‐based ceramic manufacturing (LCM) has become feasible [11, 12, 13]. Baumgartner et al. found that restorations made from lithium disilicate by LCM exhibited satisfactory printing accuracy and mechanical properties, and can be produced in the same day [14]. Unkovskiy et al. demonstrated the first clinical application of LCM‐printed dental crowns in the front region by a severely worn dentition and reported a satisfactory marginal adaptation [15]. The feasibility of LCM‐manufacturing of non‐prep veneers with a thickness in the order if 0.2 mm was demonstrated by Schweiger et al. in in vitro environment [16]. However, the topical literature lacks any clinical reports regarding the use of LCM‐made ceramic non‐prep veneers. The present clinical case presents initial experience with the clinical application of LCM‐manufactured non‐prep lithium disilicate veneers in the frontal region.

## 2 Clinical Case

**FIGURE 1:**
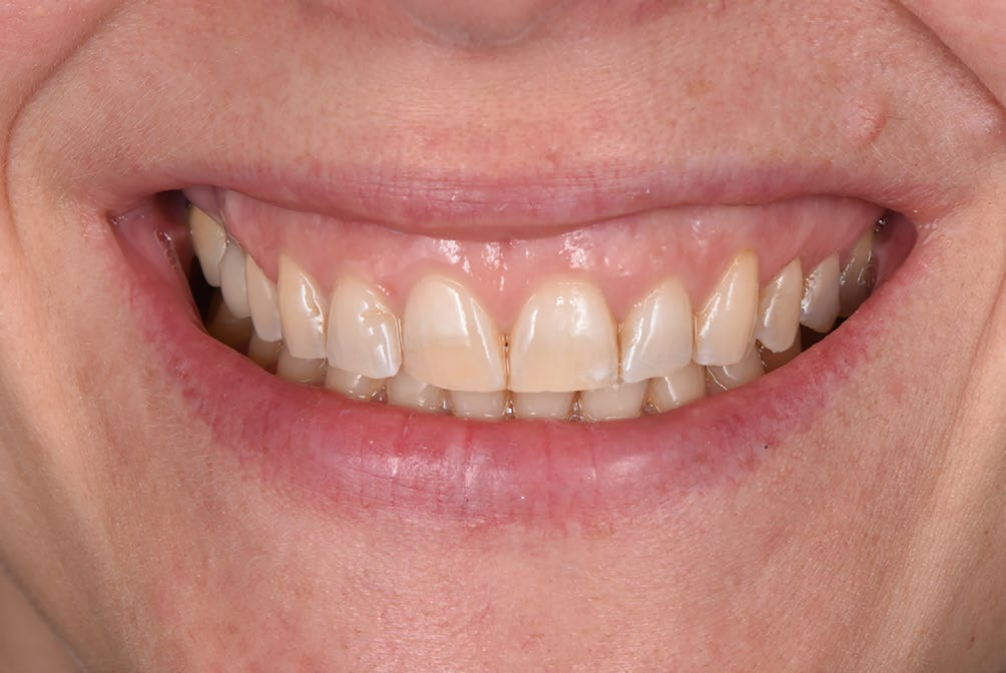
Initial situation of a female patient with worn maxillary anterior teeth.

**FIGURE 2:**
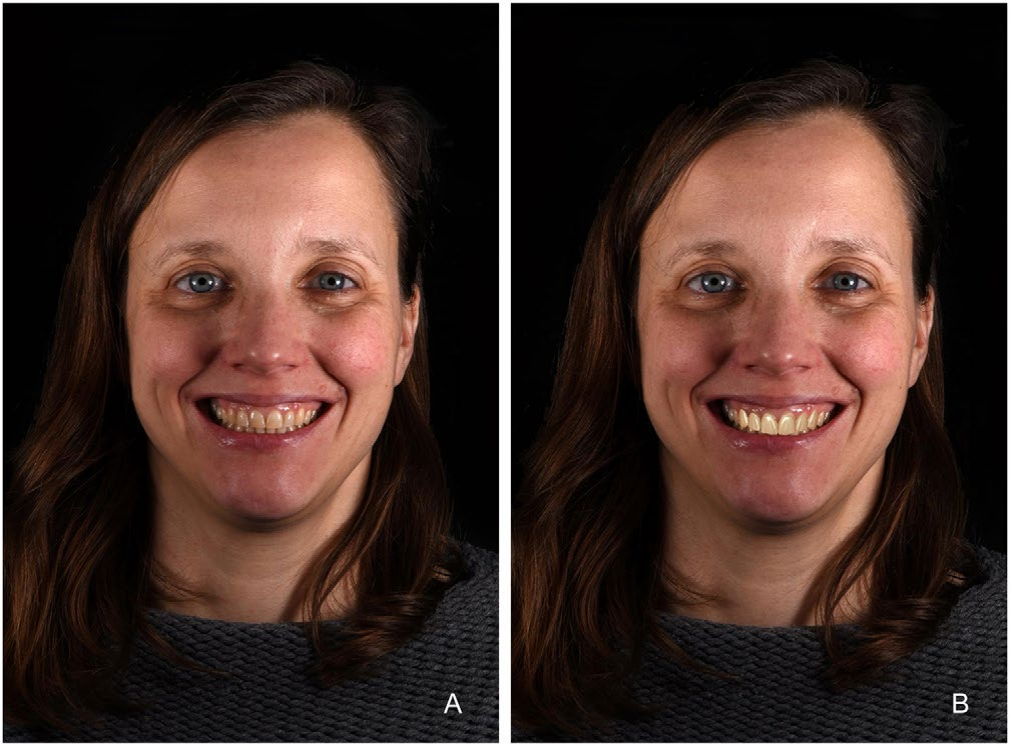
(A) Initial situation; (B) 3D smile design with the adjusted length‐width ratio of maxillary anterior teeth.

**FIGURE 3:**
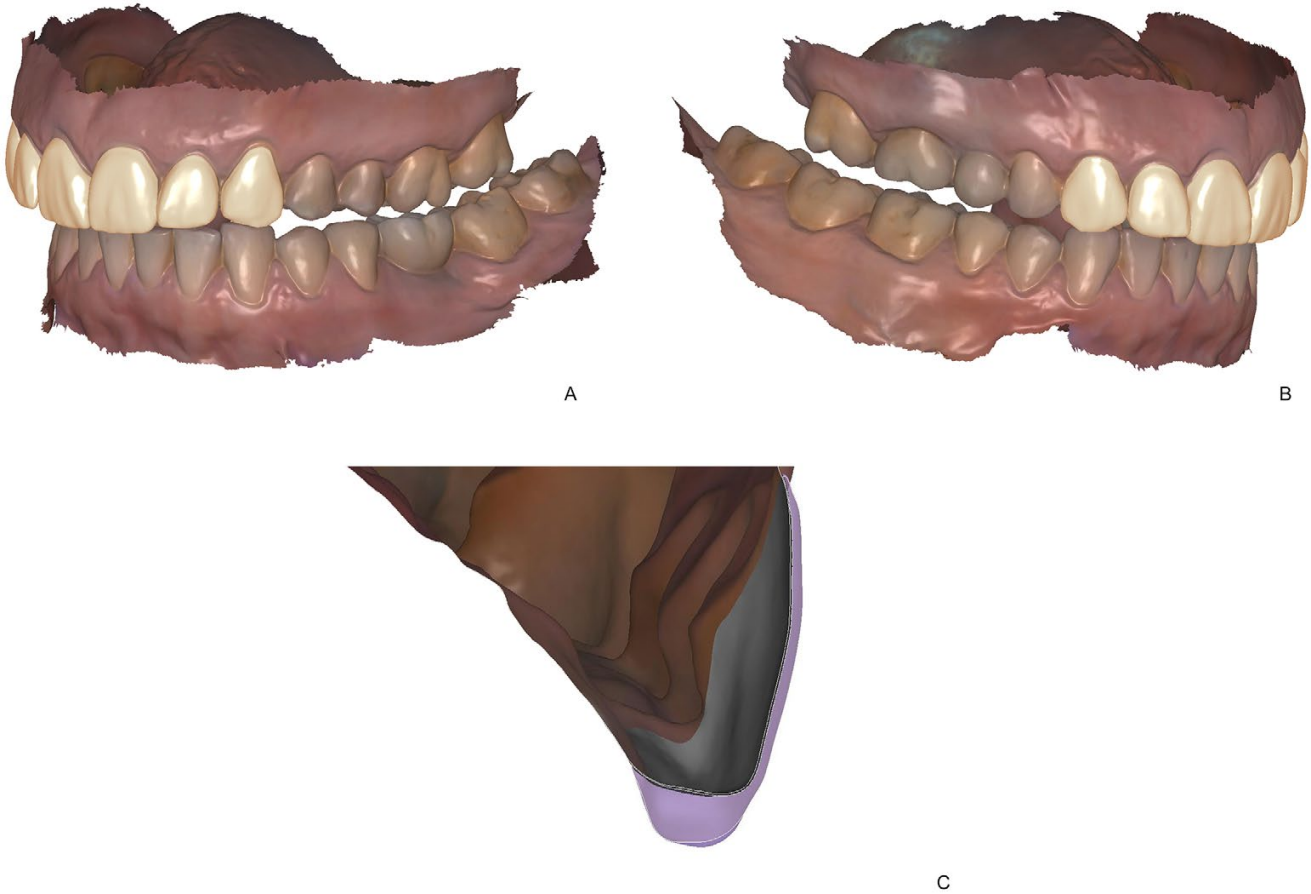
(A) The final design of six non‐prep veneers in the maxilla; (B) canine guidance was adjusted; (C) the cross‐section view of an ultra‐thin non‐prep veneer with as low as 0.1 mm restoration thickness.

A female patient with severe abrasion of the anterior maxillary teeth was referred to the department of prosthodontics at the Berlin University Hospital Charité and gave her informed written consent for participation in this trial. The patient's chief compliant was the reestablishment of the length‐width proportion of the frontal upper incisors, as it compromised her smile esthetics (Figure 1). No reestablishment of vertical dimension of occlusion (VDO) was needed, as the rest of dentition was not worn in such a severe extent. The patient's dentition was digitized with an intraoral scanner (IOS) (Prime Scan, Dentsply Sirona) and exported in standard tessellation language (STL) format. Two photographs, en‐face smiling and with cheek holder, were obtained with a digital single‐lens reflex (DSLR) camera (D650, Nikon) (Figure 2A). The 3D smile design was performed using the smile creator tool in the CAD software (DentalCAD, Exocad) (Figure 2B). The length‐width ratio was changed from initially 100% to 80%. After the patient approved the digital set‐up, a final restoration design of six non‐prep veneers was performed (Figure 3A–C). The cement gap was set to 0.5 mm. The restoration thickness on the labial side was 0.2 mm down to 0.1 mm in the zenith area.

**FIGURE 4:**
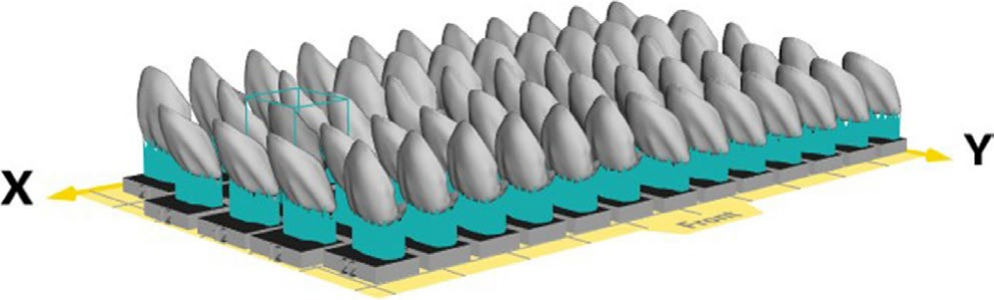
Virtual build‐platform of a CeraFab System S65 Medical with 61 ultra‐thin veneers placed ready for printing.

**FIGURE 5:**
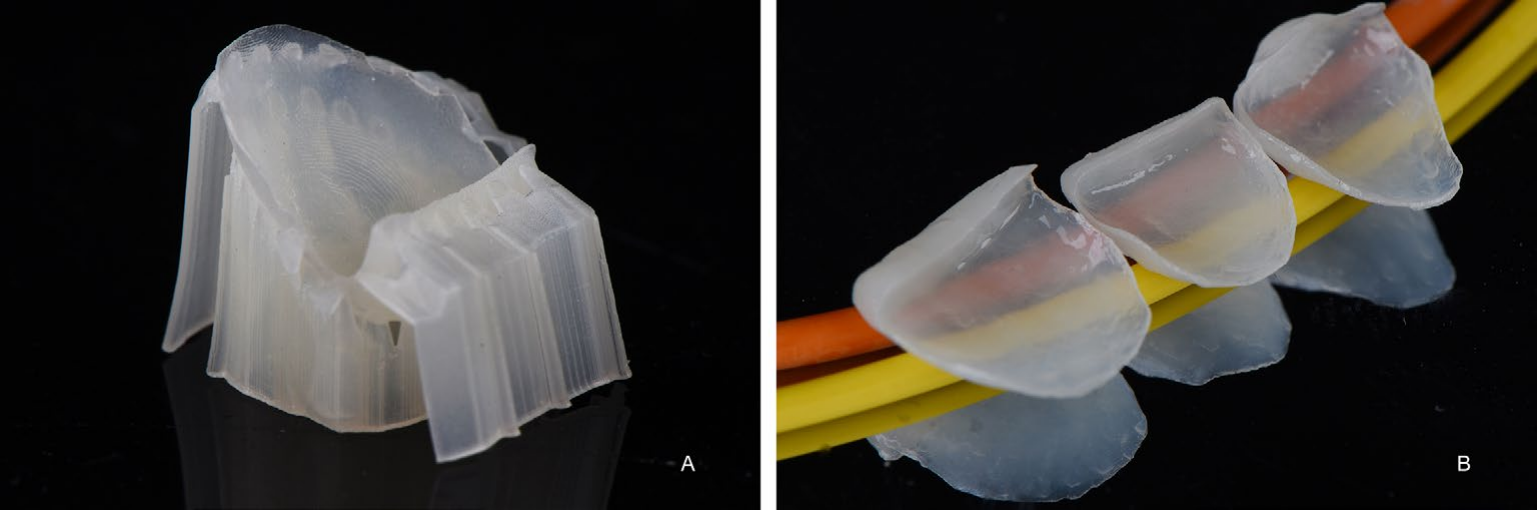
(A) The LCM‐manufactured non‐prep veneer from lithium disilicate with the supports; (B) the final view of ultra‐thin non‐prep veneers from lithium disilicate after post‐processing.

**FIGURE 6:**
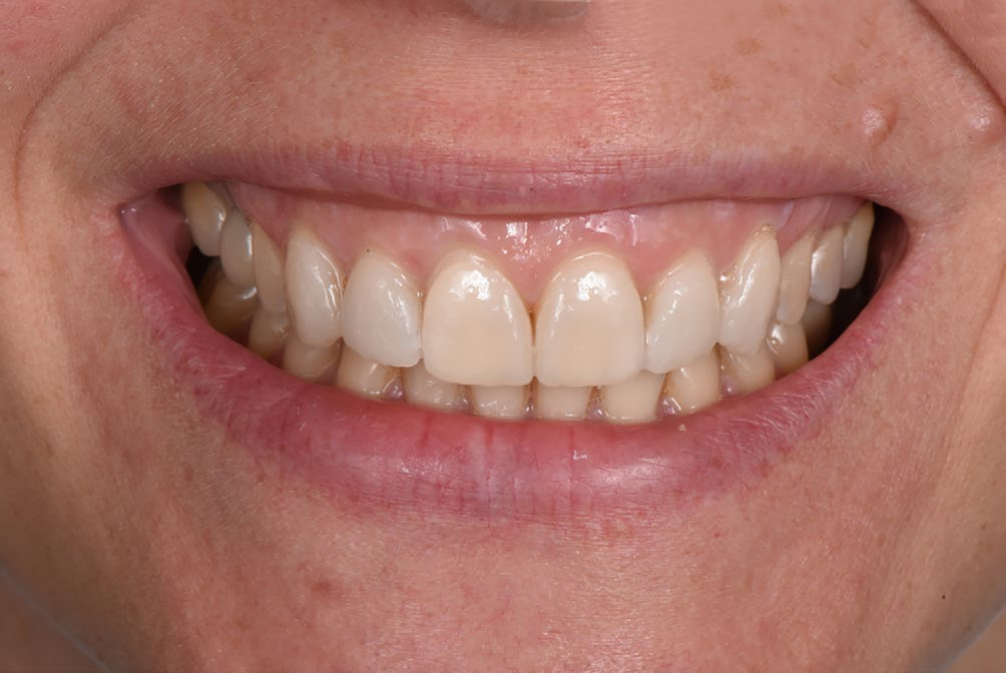
The final view of the try‐in situation of six LCM‐manufactured non‐prep veneers in the anterior region.

**FIGURE 7:**
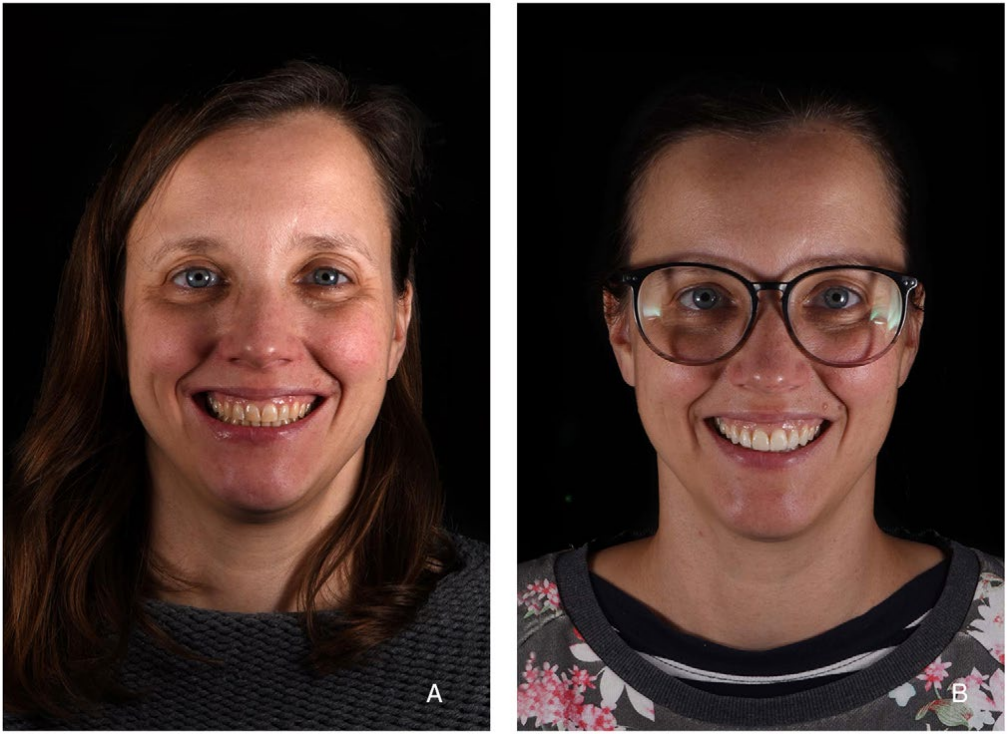
(A) Initial situation; (B) the six LCM‐manufactured non‐prep veneers in situ.

The STL data of six veneers were printed with an LCM printer (CeraFab System S65 Medical, Lithoz). The used printing parameters can be found in Table 1. The support structures were placed on the labial or incisal surface in the nesting software (Deskartes 3D Data Expert, Deskartes) (Figure 4). After printing, the LCM‐printed lithium disilicate green bodies were cleaned from excess slurry (CeraCleaning Station Ultra, Lithoz GmbH) with a cleaning fluid (LithaSol 80; Lithoz GmbH); then debinded in a furnace (Nabertherm L9/11 BO, Nabertherm) at 430°C with a dwell time of 5 h to remove all polymeric binder. The resulting parts were sintered in a furnace (Programat P510, Ivoclar Vivadent AG) at a temperature of 900°C with a dwell time of 1 s. Finally, the supports were removed with a dental technician handpiece (Perfecta 900, W & H) (Figure 5A,B). The working cast was printed using direct light processing (DLP) 3D‐printer (3Demax, DMG, Hamburg, Germany) with the model resin (Dental Peach, Harz labs, Riga, Latvia). Each veneer was tried‐in on the model. The veneers were stained (e‐max Ceram, Ivoclar Vivadent AG) by a master dental technician. The six non‐prep veneers were tried‐in on the patient using a try‐in glycerin gel (Variolink Esthetic Try‐in paste, Ivoclar Vivadent) (Figures 6 and 7A,B).

**TABLE 1:** Printing parameters for Lithography‐based Ceramic Manufacturing on a CeraFab System S65 Medical for lithium disilicate non‐prep veneers.

Material	Lithium disilicate (generic color, 45 Vol% solid loading)
Layer thickness	60 μm
Number of layers	291
Layer time	36 s
Runtime for whole print run	2.91 h
Exposure energy starting layers	500 mJ/cm^2^
Exposure energy general layers	300 mJ/cm^2^
Lateral (XY) shrinking compensation	1.254
Build direction (Z) shrinking compensation	1.288
Z curing depth compensation	On
Z curing depth compensation layers	3
Contour offset	0 μm
Support structure thickness	400 μm
Vat type	UHC with CeraVat F
Cleaning fluid	LithaSol 80
Wetting	Disabled

## 3 Discussion

Recently the 3D‐printing of definitive dental restorations became possible [15, 16]. The additive way of manufacturing enables the production of thin restorations, which in its turn helps to preserve more tooth tissue [17]. Recently there has been a trend toward 3D‐printing of hybrid resin‐ceramic restorations, which seems to be a viable clinical option [18]. Prause et al. reported the satisfactory clinical outcome over 12 months of clinical service [19].

The present proof‐of‐concept case demonstrates the clinical application of 3D‐printed non‐prep ceramic veneers in a digital workflow. The LCM technology has previously been shown to produce accurate parts with the maximum deviation of 50 μm to the initial CAD‐data [14]. The internal adaptation of LCM‐manufactured restorations was reported to be in the range of 100 to 20 μm, which correlates with the accuracy of the heat pressed and milled restorations and was regarded to be clinically sufficient [20, 21]. The present 3D‐printed ceramic non‐prep veneers demonstrated excellent fit both on the model and clinically.

Schönherr et al. described the frame‐like supporting structure to be optimal for LCM, which was also used in the present case [22]. Such kind of support, however, requires more time for removal and results in intense manual post‐processing by a dental technician. The present restorations were printed in 45° build angle, which is generally considered to be optimal for such kind of geometry using resin‐ceramic materials [23, 24]. Further investigation of the optimal build angle for sole ceramic manufacturing should be investigated in the future.

With regards to production time, up to 61 ultra‐thin veneers can be placed at a single build platform of a CeraFab System S65 Medical (Figure 4). The print run of a full platform takes slightly less than 3 h.

The manual post‐processing time per each object can be conducted in around 2 min including placing in the furnaces. For this reason, the time effectiveness seems to be significantly higher compared to the conventional injection‐molding manufacturing of lithium disilicate restorations. Furthermore, multiple restorations can be 3D‐printed and post‐processed in the furnace at the same time, whereas in the conventional workflow a dental technician can manually process only one restoration at a time [25]. The time needed for individualization and staining the post‐processed part is not included in this time, as its highly dependent on the experience of a dental technician or dentist.

The future perspective of additive manufacturing of lithium disilicate may also consider multicolor 3D printing. This would be a game changing feature, as it would eliminate the individualization through the dental technician and would be one more step toward fully chairside lithium disilicate manufacturing.

With regards to price of the final 3D printed lithium disilicate restoration, the cost estimation is based on the material price, the machine depreciation and the costs for manual labor post‐processing. If compared to subtractive manufacturing the savings on expensive tools makes 3D printing especially attractive.

The current clinical case did not consider the mechanical and adhesive properties of 3D‐printed restorations. Notably, non‐prep restorations are highly delicate, underlining the clinical significance of their flexural properties. Consequently, future research should consider evaluations of flexural and bonding strength, in conjunction with surface characterization of the LCM‐manufactured lithium disilicate. The clinical and technical strategies also present novel perspectives for in vivo testing of additively manufactured ceramic restorations, given their future clinical approval.

## 4 Conclusion

This proof‐of‐concept clinical case demonstrates the feasibility of clinical application of 3D‐printed ultra‐thin non‐prep lithium disilicate veneers for esthetic smile enhancement. The LCM technology provided rapid manufacturing of thin restorations and may be considered in the future as an alternative for subtractive manufacturing.

## Consent

Informed written consent for participation in this trial was obtained prior to intervention. An additional written informed consent for publication of clinical photographs was obtained prior to submitting this manuscript.

## Conflicts of Interest

The authors declare no conflicts of interest.

## Data Availability

The data that support the findings of this study are available from the corresponding author upon reasonable request.
